# Examining motivation within the context of acculturative stress: a systematic review

**DOI:** 10.3389/fpsyg.2025.1734112

**Published:** 2026-04-07

**Authors:** Arianna Haviv Zehner

**Affiliations:** Institute of Educational and Developmental Psychology, Technical University Dresden, Dresden, Germany

**Keywords:** acculturation, acculturative stress, international students, language, motivation

## Abstract

**Introduction:**

Language proficiency is a well-established predictor of acculturative stress (AS). However, focusing predominantly on proficiency may overlook other important dynamics in how language relates to AS. Shifting attention toward motivational factors offers a complementary perspective, allowing for a deeper exploration of how individuals engage with language learning and use within the acculturation process.

**Methods:**

This systematic review is the first to investigate the relationship between motivation and acculturative stress in the last decade (2014–2025), across nine major data banks and Google Scholar.

**Results:**

Results demonstrate that while seven motivation constructs (including academic motivation, motivation to adapt, and motivation to study abroad) are measured in relation to acculturative stress, language learning motivation is very underrepresented. This is an unfortunate oversight, as language has been proven elemental in the acculturation process.

**Conclusion:**

Although acculturative stress and motivation are well-researched independently, there is little research combining them in the quantitative sense. This review exposes a literature gap and elaborates on why language learning motivation could enhance acculturative stress literature.

## Introduction

Acculturation is the ongoing interaction between an individual (or group) and a new host-culture, country, or population (e.g., Berry et al., 1987). Acculturative stress (AS) is often defined as the reaction to the acculturative process: the stress of adapting to a host culture (Berry, 1970) and the stressors associated with being an immigrant or ethnic minority (Borrego et al., 2019). Acculturative stress is a measurable phenomenon; there are over 30 tools to measure acculturative stress (Rudmin, 2009). The Acculturative Stress Scale for International Students (ASSIS) is the most widely used (Çimşir and Kaynakçı, 2024) and was developed by Sandhu and Asrabadi (1994). Literature consistently attests to the inverse relationship between language proficiency and acculturative stress (AS) – the more proficiency, the less AS (i.e., Andrade, 2006; Benzie, 2010; Constantine et al., 2004). However, this insight relies on how proficiency is measured. Standardized approaches to measuring language aptitude have been utilized – such as the Test of English Language Fluency (TOEFL) — which is commonly given to international students. Non-standardized (self-reported) tools, such as the Self-Reported Fluency English Survey (SFRES) are also commonly administered in ASSIS literature (i.e., Rajab et al., 2014; Thomson and Esses, 2016; Wang et al., 2017). Developed by Yeh and Inose (2002), this 3-Question survey essentially asks how comfortable respondents feel speaking English (but could be adapted for any host language).

Despite their utility in gauging language skill, these forms of measurement do not provide any insight into language learning motivation (LLM) or overlapping motivation constructs (such as cultural or integration motivation) which may include perceptions of the host society, willingness to engage, or personal goals (Gardner and Lambert, 1972). These constructs could potentially offset acculturative stress.

The fixation on acculturative stress has led to neglect of “motivations of utility” (Rudmin, 2009). By placing disproportionate emphasis on perceivably stressful circumstances, we potentially ignore the other side of the story: the will to overcome. Motivational aspects are central to understanding stress, as motivation often occurs *with* the stressor (Lazarus and Folkman, 1985); although stress is not always considered as an antecedent of learning motivation, the transactional theory of stress provides support for this relationship (Lazarus and Folkman, 1985). More insight into the effect of language on acculturative stress can be disclosed through shifting focus away from measuring proficiency and more toward various motivation constructs. There are several overlapping motivation constructs which intersect with — and potentially contribute to — our understanding of language motivation. For example, the motivation to adapt (Masgoret et al., 1999). As Rudmin (2009) noted, acculturation literature is disjointed from second-language learning research, even though language learning plays a role in the acculturation process; only 8% of PsycARTICLES articles mentioning “acculturation” also mention “second-language.” It is well-established that *motivation* is a powerful component of language learning (Dörnyei, 1998; 2001; Lăpădat and Lăpădat, 2023), and should therefore be investigated with respect to AS. This insight begs the question: to what extent are motivation constructs represented in ASSIS literature? Therefore, the objective of this systematic review is to investigate which motivation constructs are investigated and *measured* in context to AS.

## Problem statement

The relationship between acculturative stress and language proficiency has been measured — established methods already exist to quantifiably assess this dynamic. Despite the well-established transactionary relationship between stress and motivation in general, the specific relationship between motivation and acculturative stress remains unclear. This is especially true regarding language learning, despite that language plays a fundamental role in the acculturation process. There are several potential reasons for this oversight: First, measuring language learning motivation could pose a unique challenge to ASSIS researchers who study international students. In instances where English is the academic language of instruction but not the host language, this could mean investigating (and perhaps even comparing) the motivation behind at least two languages. Second, researchers who choose to study international students may prioritize acculturative stress from the academic perspective rather than the cultural – placing more emphasis on academic motivations rather than cultural or integrative. In short, researchers may choose between life on the campus, within the host society, or a combination.

In an attempt to recover the “motivation of utility,” this systematic review seeks to investigate which motivation constructs have been researched in the past decade with ASSIS and to understand how motivation is measured relative to stress. As language motivation potentially shares theoretical overlap with other forms of motivation (such as the will to adapt, or study abroad motivation), this review seeks to uncover any studies which have addressed any motivation construct in tandem with ASSIS.

## Method

### Eligibility criteria

This review focuses exclusively on the Acculturative Stress Scale for International Students (ASSIS). Aside from being the most widely used scale for measuring acculturative stress (Çimşir and Kaynakçı, 2024), there are additional justifications: Unlike other acculturative stress scales such as the Acculturative Stress Scale for Chinese Students (ASSCS; Bai, 2016) and the Acculturative Stress Scale for Pakistani International Students (ASSSIS; Bashir and Khalid, 2020), the ASSIS it is not specific to any particular demographic. This allows for more diverse and general usage, compared to other scales which are geared toward children [i.e., Children’s Acculturation Report-Assessment (CAR-A); Park-Taylor, 2004], adolescents [Adolescent Stress Measure for Asian Americans (ASMAA); Kim-Bae, 1999], parents [Southeast Asian Parenting Stress Scale (SAPSS); Hayashino, 2003], or particular nationalities.

Additionally, this review focuses on ASSIS because it uses a more dimensional approach to measuring AS (with 7 factors), whereas other scales rely on as few as four [such as the Multidimensional Acculturative Stress Inventory (MASI; Rodriguez et al., 2002)]. This comprehensive rationale focuses on ASSIS for its popularity, diverse participant pool, and broader psychological dimensions.

To be included in this review, papers must have adhered to the following criteria: (1) Studies must have administered the ASSIS (either partially or completely) *and* (2) have attempted to measure motivation. Studies which have measured motivation *in any capacity* were included, as it is possible that LLM was measured through the virtue of a different motivation measurement (for example, cultural motivations, study abroad motivations, and the like). Therefore, to prevent overlooking LLM, all forms of motivation (so long it had been measured in conjunction with the ASSIS) were accounted for. Studies which attempt to measure motivation but do not incorporate ASSIS were disqualified. Similarly, publications which had implemented ASSIS but had not attempted to measure motivation were omitted. There is no limit to the number of scales one could utilize in a study, so long as ASSIS was one of them, and motivation was assessed through *any* measurement tool. Articles must have been published in English and published in the last decade (2014–2025). The search began in January of 2025 and concluded on March 1, 2025.

### Data banks

The following research data banks were searched by two reviewers independently: (1) APAPsycArticles, which is the full text database of the American Psychological Association (APA). It contains more than 200,000 articles from 135 peer-reviewed journals from the APA and other publishers. It encompasses 30 data banks. (2) APAPsycInfo, which is a separate but related data bank which includes approximately 2,500 journals across a wide spectrum of interdisciplinary fields, including psychology and educational studies. (3) The Social Sciences and Humanities journal collection (SSH Library) was searched, which includes more than 1,500 journals from the publishers Taylor & Francis, Routledge and Psychology Press. (4) The Hogrefe data bank, which encompasses over 60 journals, all of which focus on Psychology or related themes. (5) peDOCS, which is a bank for educational scientific literature currently composed of about 28,800 publications, (6) SCOPUS, (7) Academic Search Premier (via EbscoHost), (8) Web of Science, (9) The Taylor & Francis The Social Sciences and Humanities journal collection (SSH Library), and (10) Google Scholar, which is a free, popular, and publicly available academic search engine for literature of any kind.

### Search strategy

**Figure 1:**
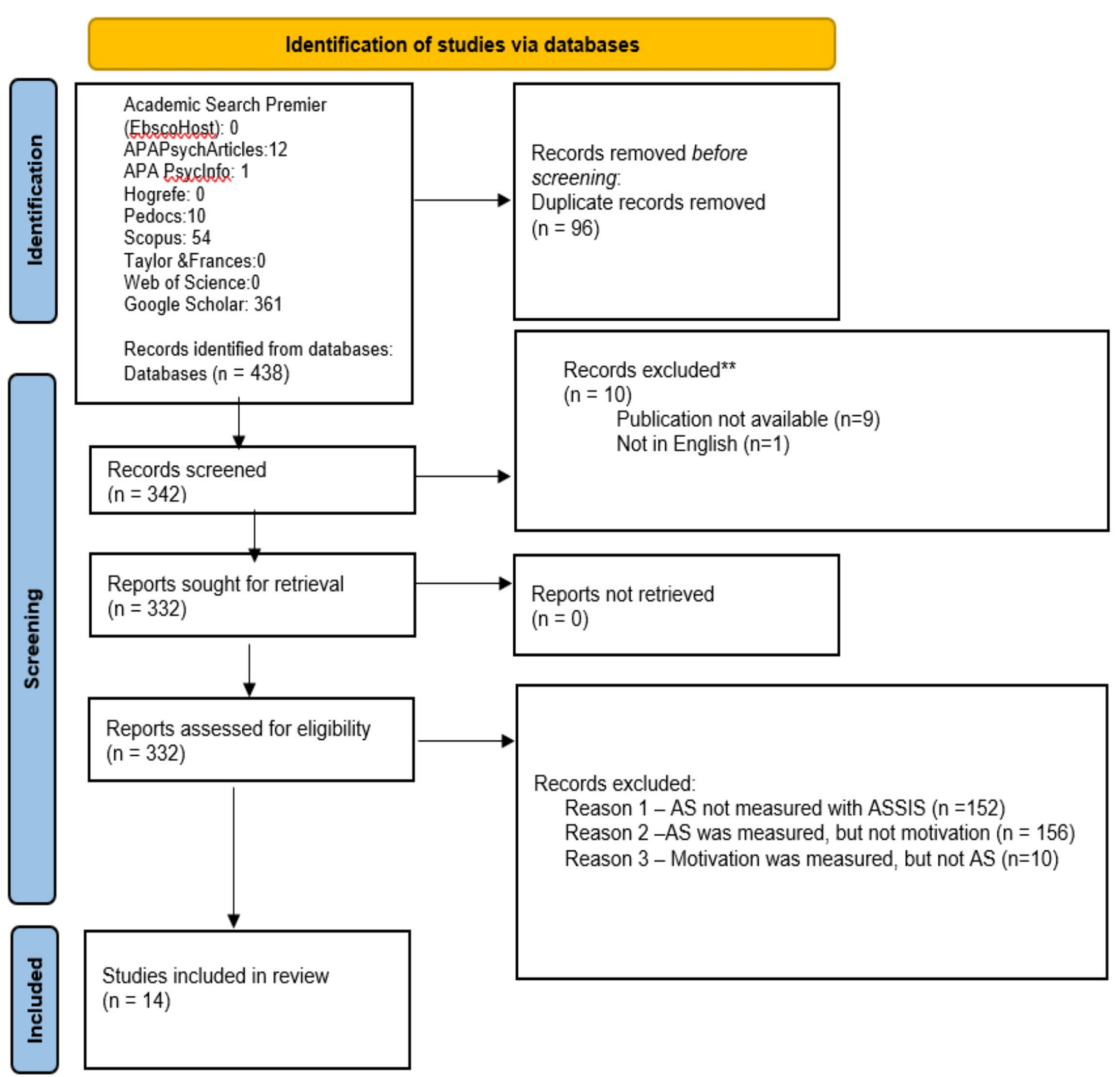
PRISMA flow chart of records.

Both reviewers used the same search terms: “acculturative stress scale for international students” AND “language” AND “motivation.” The specific term “language motivation” would limit the scope to such an extent that it would be difficult to see what *other* types of motivation are present, if not language motivation. Keeping “language” and “motivation” as independent search terms ensures that each will be present in the publication, either in their own right, or in conjunction with each other. Regarding acculturative stress: The key term “ASSIS” was not used to prevent confusion with other terms which contain “assis-” or potentially other acronyms. Additionally, the search term “Acculturative Stress Scale” itself is not specific enough, as there are dozens of other scales for AS aside from the ASSIS, which this review is focused on. Additional parameters limited the search to only include literature that had been published in the last decade (January 1, 2014 – March 01, 2025). Thus, the publications which included all three of the key phrases *and* were published between 2014 and 2025 were selected for review. These initial parameters yielded a total of 438 publications across the 10 sources. Figure 1 cited a PRISMA standard flow chart depicting the selection process.

### Selection strategy

As a next step, the two reviewers were independently tasked with screening the compiled literature, namely: One, to remove duplicates (the same publications which were found in separate data banks). After this step, 342 unique publications remained – 10 were discarded for being unavailable, 332 remained for screening. Screening these 332 publications, the two reviewers needed to confirm (for each remaining publication) that the ASSIS was used to measure AS. Similarly, reviewers needed to confirm that motivation was also measured. This step is crucial, because although the papers contain the key terms, it is no guarantee that acculturative stress (and/or motivation) are measured, as opposed to merely being mentioned or discussed. To be included, each publication should clearly state whether motivation is to be measured or if it is included as an item on a relevant scale. This guideline aims to prevent the subjective interpretation or over-extension of “motivation” into other similar territories (such as self-efficacy, resilience, etc.).

For example, it cannot be assumed that if a study measures “contact with locals” that motivation is measured (although they are inherently related). “Motivation to adapt” would be included for review, whereas a scale measuring “psychological adaptation” but not explicitly the motivation, would not count. If the paper did not satisfy both criteria (measurement of AS and motivation), it was rejected. Each paper could only be rejected/accepted with unanimous agreement from both reviewers. If motivation was measured, reviewers noted whether language motivation specifically was measured, and if not language motivation, to note what kind of motivation was measured in relation to acculturative stress. All motivation constructs are noted, because they may relate to language motivation; it may be captured as a subcategory of another motivation or captured as items in a related measurement tool/survey.

## Results

### Data items

Of the 332 papers screened, 14 had measured motivation in conjunction with ASSIS, including 7 types of motivation. Roughly 4.2% of ASSIS literature accounts for motivation. Table 1 displays the 14 papers and which form of motivation was measured.

**Table 1:** Literature measuring both motivation and acculturative stress (*n* = 14).

Author(s) and year	Title	Type of motivation	Measurement tool
Anderson and Guan (2018)	Implicit acculturation and academic adjustment of Chinese sojourners in Australia	Academic Motivation (subfactor of Academic Adjustment)	Academic Adjustment Scale (AAS; Anderson et al., 2018)
Atteraya (2021)	Acculturation stressors and academic adjustment among Nepalese students in South Korea	Student Motivation (adapted SACQ)	SACQ (Baker and Siryk, 1986)
Bastien et al. (2018)	Striving for success: Academic adjustment of international students in the US	Motivation to Adapt	SACQ (Baker and Siryk, 1989)
Cheng (2017)	Religion/spirituality and acculturation stress among international students	Intrinsic Religious Motivation	Religion and Spirituality Scale (Hoge, 1972; Sheridan, 2016)
Le Hong and Hsu (2024)	Effects of cultural distance and discrimination on student tourists in Taiwan	Travel Motivation	Perceived Cultural Distance (Fan et al., 2023)
Mucsi (2021)	Study abroad motivations, satisfaction and loyalty	Motivation to Study Abroad	Researcher-developed
Sabrina, FIONA (2014)	Acculturative stress, supports and achievement motivation among Indonesian students	Achievement Motivation	Achievement Motivation Scale (Ye and Hagtvet, 1992)
Sakamoto (2015)	Motivation, second language learning, and stress in international students	Language Learning Motivation	LLOS (Noels et al., 2000)
Van Froelich (2018)	Mediating influence of acculturation on student adaptation	Motivational Influences on Adaptation	Willingness to Communicate Scale (Shao and Gao, 2016)
Viswambharan (2023)	Acculturative stress, academic motivation and personality among Indian students	Academic Motivation	AMS-College Version (Vallerand et al., 1992)
Wahba (2016)	Constructivist learning environments and outcomes for international students	Motivation to Learn (Constructivist)	Not reported
Wang et al. (2023)	Acculturative stress, acculturation, hope and cultural intelligence	Motivation (subfactor of CQ)	Cultural Intelligence Scale (Ang et al., 2007)
Wang et al. (2025)	Longitudinal study of acculturative stress and depression in minority students	Motivation (subfactor of CQ)	Cultural Intellgence Scale (Ang et al., 2007)
Yueyue et al. (2022)	Intercultural adaptation in Sino-American dual-degree programs	Motivation and Expectations to Study Abroad	Tan and Liu (2014) Adaptation Scale

**Figure 2:**
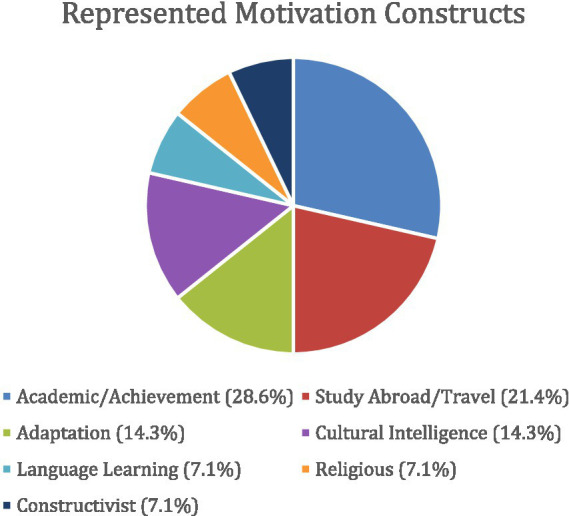
Motivation representation among ASSIS literature (*n* = 4).

Figure 2 displays the classified representation of various motivation constructs. Assessments of academic motivation and motivation to study abroad/travel are more represented, whereas language motivation, religious motivation, and constructivist motivations were the least represented. However, it is clear that motivation constructs in general are rarely utilized with ASSIS. Despite the role that language learning plays in the international classroom and in the study abroad lifestyle, neither academic motivation nor study abroad motivation had accounted for language learning.

## Discussion

This review intended to establish acculturative stress as an important context through which motivation can be measured. Considering the unique role of foreign language learning in the acculturative process, one goal of this review was to investigate whether language motivation (either directly or by proxy through other related constructs) is measured in relation to acculturative stress. Across nine major databases and Google Scholar, this review was able to uncover 14 articles and 7 different types of motivation. Although various constructs pertain greatly to language learning (i.e., cultural intelligence, adaptation, study abroad, academic motivation), only *one* paper in the last decade explicitly measures both language motivation and acculturative stress, and that was Sakamoto (2015). Although Sakamoto (2015) had administered the ASSIS (measured acculturative stress) and also effectively measured motivation, the goal of Sakamoto (2015) was more oriented to the relationship between language motivation and proficiency, rather than the link between language motivation and AS directly. Sakamoto (2015) suggests that stress was a better predictor of language skill than motivation, which supports prior literature attesting to the positive relationship between stress and motivation (i.e, Lin et al., 2020). Despite the underrepresentation of language learning directly, the six other motivation constructs still provide valuable insight into which forms of motivation are represented with ASSIS:

Motivation to Adapt: Arguably one of the most relevant to language learning motivation is the motivation to adapt. As measured by Van Froelich (2018) through the Willingness to Communicate scale, the fundamental desire or interest in communicating with locals is paramount to foreign language learning, especially when it comes to fostering adaptation (Van Froelich, 2018; Shao and Gao, 2016). It has long since been acknowledged that the willingness to communicate is an indicator of language learning success (e.g., Yang et al., 2022). While willingness to communicate itself would not be eligible for review, it is under the umbrella of “motivation to adapt,” which was ultimately sought by the reviewed publication.

Academic Motivation: The relevance of academic motivation within ASSIS is established among the context of international students abroad and the need to investigate their academic prowess while in a foreign country. It was found that high academic motivation to attend college resulted in less acculturative stress. Viswambharan (2023) acknowledges language as a factor of cultural adjustment, noting: “the intensity of the acculturative stress depends on the extent to which the host culture and the individual’s personal culture are similar or dissimilar, which includes language and other variables […].” Anderson and Guan (2018) had focused (in part) on academic motivation, as well as academic lifestyle and the role of identity among Chinese international students in Australia. They had found academic motivation to be a potentially helpful barrier of acculturative stress, as it potentially preserves a sense of Chinese identity, and helps the students to focus on their goals, rather than succumbing to culture shock. Moreover, the temporary nature of studying abroad has removed motivation to adapt into the host culture (including motivation to learn the language), whereas academic expectations persist regardless of the duration of the time abroad.

Achievement Motivation was also represented in the pool of literature and was classified as akin to academic motivation. Sabrina, FIONA (2014) investigated achievement motivation among Indonesian university students at Malaysian universities and also found a correlation between the motivation to achieve goals was negatively correlated with acculturative stress. Specifically, Sabrina, FIONA (2014) had implemented the Ye and Hagtvet (1992) Achievement Motivation Scale which was divided into two subscales: One for motivation to succeed and the other for motivation not to fail, which had also been based on the construction from Atkinson and Feather (1996).

Motivation to Study Abroad was researched by Mucsi (2021) as a mediator of culture shock (as measured through ASSIS). The underpinning of this study is related to the personal interest that language learning entails. The author revealed that indeed having an interest in the culture, the host community, the language, are good predictors of having lower acculturative stress. As indicated by the result of this study, language barriers can develop into homesickness (Mucsi, 2021). The author also echoes similar sentiments regarding the importance of learning the local language: “lack of knowledge of the local language, the type of foods and available accommodation were often mentioned by students as a factor of instant shock[…] students with motivations connected to gaining life experience abroad or learning about the country (and its culture) were more satisfied than students driven by social pressure or adhering to a heavily restrained or cost saving lifestyle abroad” (Mucsi, 2021). These results further attest to how assessing language learning motivation (either additionally or independently) could foster a greater understanding of life abroad for international students.

Religious Motivation was also represented by only article (Cheng, 2017), and is therefore just as underrepresented as language motivation in the ASSIS corpus. Cheng (2017) had explored the relationship between religious spirituality and acculturative stress among international students in Western Massachusetts. The author implemented three scales: ASSIS, the Religion and Spirituality Scale (Hoge, 1972; Sheridan, 2016), and the Intrinsic Religion Motivation Scale (Hoge, 1972). As the study focuses on international students, language was addressed: Participants were asked whether English was their native language (31.6%) and whether they had learned English in their home countries (65.8%). The author speculated a language barrier as a potential cause of high drop-out rate while simultaneously confirming the high acculturative stress of religious participants.

### Limitations

As this review focuses specifically on motivation measured with the ASSIS, it cannot attest to the extent to which motivation is paired with any other acculturative stress scale. Within the scope of ASSIS literature, this review cannot attest to the extent to which motivation is accounted for prior to the year 2014. In the literature pool, measurements of various motivation constructs were occasionally paired with ASSIS, however, there are also similar themes which are of relevance. The first example is self-efficacy: Although self-efficacy can be very relevant to the role of motivation within acculturative stress, articles which had measured self-efficacy (but not explicitly motivation) — such as Yan (2024) — could not be included in the review for fear of conflating the terms or extending the definition of motivation too liberally. Self-efficacy is often defined as the belief in one’s ability to take action, meet task demands, and obtain desirable outcomes for oneself (Bandura, 2005; Wood and Bandura, 1989). In regard to intercultural adjustment, self-efficacy is one’s belief that they are able to manage intercultural challenges and function within the host culture or society (Black and Gregersen, 1991; Fan and Mak, 1998; Hua et al., 2020), which naturally includes language learning.

The second example is “language confidence”: Wu et al. (2023) was not included in the review but remains relevant; the authors had implemented the ASSIS with other scales *similar* to language learning motivation, including confidence to use Cantonese and anxiety in using Cantonese. The authors claim that the results attest to the *likely* inverse relationship “between Cantonese anxiety and language learning motivation” (Wu et al., 2023), although it cannot be confirmed, as language learning motivation was not investigated specifically. Notably, Yu et al. (2019) (who was also not eligible for review) had echoed similar sentiments: “coupled with the lack of motivation to learn Cantonese among the English-speaking international students, there is a lack of learning opportunities. Due to this combination of factors, it is difficult to enhance Cantonese skills simply through time as suggested by the cultural learning theorists” (Yu et al., 2019). As such, they provide support for why language learning motivation and acculturative stress should be researched in relation to one another.

A final example of theme-overlap pertains to adaptation and integration: Yang et al. (2023) had implemented the Perceived Discrimination factor from the ASSIS but had measured cross-cultural adaptation and integration strategy rather than language motivation. Still, it should be noted that the authors cite self-determination theory as a key source which would influence their motivation and behavior during integration or adaptation (Deci and Ryan, 1985). The interest in engaging with locals or learning the host language is relevant to this theme, however, the items from their survey are unfortunately not readily available, and it cannot be confirmed or assumed that language learning was a part of this study.

### Suggestions for future research

Despite that motivation and AS are well-researched fields independently, there is very little attempt to bring them together in a quantifiable sense. As is evident, the measurability of various, inter-related forms of motivation have been established. Yet, various motivation constructs still remain a very small percentage of ASSIS literature of the past decade. Motivation remains something more often discussed than measured. As the context appears conducive (based on mutual themes of intercultural communication, integration, and the like). Motivation constructs of various forms should be more often incorporated with ASSIS to provide more of a comprehensive understand of international student/migrant attitudes.

While the present review exposes that language learning motivation is quite overlooked within the context of acculturative stress, future research should address this by assessing the relationship between language motivation and AS. Specifically, this means attempting to *measure* the effects of acculturative stress on language motivation or vice versa.

## Data Availability

The set of articles collected for this review can be found in this public repository and is open access: 10.5281/zenodo.17456922.
